# Investigation of Frequency-Specific Loudness Discomfort Levels in Listeners With Migraine: A Case–Control Study

**DOI:** 10.1097/AUD.0000000000001339

**Published:** 2023-02-15

**Authors:** Angeliki Mourgela, Michail Vikelis, Joshua D. Reiss

**Affiliations:** 1 Queen Mary University of London, Bethnal Green, London; 2 Glyfada Headache Centre.

**Keywords:** Auditory test, Migraines, Sound hypersensitivity

## Abstract

**Objectives:**

Hypersensitivity to auditory stimuli is a commonly reported symptom in listeners with migraine, yet it remains relatively unexplored in research. This study aims to investigate loudness discomfort levels in listeners with migraine, while identifying the frequencies most affected by the phenomenon.

**Design:**

To achieve this, the study compared just audible level and loudness discomfort level ranges between participants with and without migraine from the United Kingdom, Greece as well as the participant recruitment platform Prolific, across 13 frequencies from 100 to 12,000 Hz, through an online listening test.

**Results:**

Fifty-five participants with migraine and 49 participants without migraine from both countries and Prolific were included in the analysis, where threshold ranges between just audible and mildly uncomfortable levels were compared in 13 frequencies. Migraineur group participants presented significantly smaller ranges between just audible and mildly uncomfortable level, due to lower thresholds of mild discomfort in 12 of the 13 frequencies when compared with the nonmigraineur group participants. Participants taking the test during their migraine attack or aura presented a tendency for smaller ranges. In addition, participants with self-reported higher severity migraine exhibited bigger ranges compared with participants with low severity migraine within the migraineur group. No relationship between ranges and medication or migraine attack frequency within the migraineur group was observed.

**Conclusions:**

Results from the study demonstrate a tendency for the migraineur group to present lower thresholds of mild discomfort compared with the nonmigraineur group, aligning with previous studies while extending the phenomenon to more frequencies than those previously examined. Though the present study presented no relationship between ranges and medication or attack frequency, further research is required to investigate a potential link between these factors.

## INTRODUCTION

It has been reported that 52 to 82% of people with migraine have hypersensitivity to sound related to their migraine, with varying degrees ranging from mild aversion to phonophobia (Kayan & Hood 1984; Schwedt 2013). In addition, sound hypersensitivity and aversion have been widely used as one of the diagnostic criteria for migraine and its differentiation from other types of headache (Solomon & Lipton 1991; Lipton et al. 2004; Olesen 2018). Even though migraine-related hypersensitivity to sound is a highly prevalent and very commonly reported phenomenon in practice, its characteristics remain relatively unexplored, with only a few studies examining migraine-suffering listeners’ response to auditory stimuli. Results from previous studies demonstrate a tendency for listeners with migraine to present lower loudness discomfort levels compared with healthy controls (Woodhouse & Drummond 1993; Ashkenazi et al. 2009), with no audiometric abnormalities observed at the threshold of hearing. However, previous studies present limitations, such as testing for a single frequency or a limited frequency range (Main et al. 1997; Ashkenazi et al. 2009; Hamed et al. 2012; Joffily et al. 2016), using everyday sound stimuli (Tomoharu Ishikawa & Hirata 2019; Enzler et al. 2021) or sampling from a specific category of participants with migraine (Ashkenazi et al. 2009) only (e.g., episodic, or chronic, with/without aura).

The goal of this study is to identify whether listeners with migraine will present smaller dynamic ranges as a result of lower mildly uncomfortable levels (MULs) across frequencies, as well as to identify whether certain frequencies are more affected by the phenomenon. Based on previous data from related studies, we hypothesize that participants with migraine will present lower MULs compared with participants without migraine. Results from this study can further contribute to our knowledge on sound hypersensitivity and the range of frequencies affected, as well as encourage further research on specific audio adjustments for listeners with migraine. To assess our hypothesis, we investigated MULs across 13 frequencies within the range of 100 to 12,000 Hz, between adult participants with migraine and participants without migraine with no history of hearing loss. The extensive range of frequencies was selected to provide more information on the frequency dependencies of hypersensitivity, as well as to ensure the reproduction of an adequate number of frequencies, regardless of the participants’ equipment’s frequency response. To achieve this, we employed an online listening test, assessing the range between just audible levels (JALs) and MULs of sound for pure tones in the 13 frequencies mentioned above, between 2 groups of participants: participants with migraine and participants without migraine.

The use of online platforms on auditory-related listening tests has gained popularity in the recent years since they can provide researchers with easy access to a wider audience, as well as reduce the need for special training and audiometric equipment. Although web-based auditory screening can be inaccurate and prone to biases and error, it has been shown to provide an effective and more objective approach to that of self-reporting when utilized for large population studies (Bexelius et al. 2008). This study was performed through a web-based platform, recruiting participants from both the United Kingdom (UK) and Greece (GR).

## MATERIALS AND METHODS

### Ethics and Safety

Ethics approval for the study was obtained from the Queen Mary University of London Research Ethics Committee in the UK (QMERC2513a), as well as the Scientific Committee of Mediterraneo Hospital in Greece (protocol number: 341). Participants were informed about the scope of the study and were requested to confirm their understanding of the study and its requirements, as well as their agreement with the terms and conditions before taking the test, using the on-screen consent button. Participants were also reminded to keep safe listening levels throughout the duration of the study, take breaks as needed, and prompted to terminate the test in the event of pain or severe discomfort. A calibration procedure was embedded in the test to ensure the participants’ safety, as well as to improve the accuracy of the results.

### Participant Recruitment

Two main groups of participants were recruited for this study over a period of 9 months: a nonmigraineur group including participants with no migraine and no history or experience of hearing loss and a migraineur group including participants with migraine and no history or experience of hearing loss. Participants in the migraineur group were asked to confirm whether they experience or have been diagnosed with migraines and their inclusion in the migraineur group was based on self-reporting. Similarly, participants from both groups were asked whether they have ever experienced or have been diagnosed with any form of hearing loss.

UK migraineur group participants were recruited through online communities for people with migraine, the Prolific participant recruitment platform, as well as by advertising the study on the Migraine Trust. GR migraineur group participants were recruited through online communities for people with migraine on social media, as well as through the Greek Association of Patients with Migraine and Headache. Participants from the UK nonmigraineur group were recruited via the Prolific platform, social media advertising as well as the Electronic Engineering & Computer Science department of Queen Mary University of London, whereas participants for the GR nonmigraineur group were recruited just by social media advertisement.

### Exclusion Criteria for Submissions

To ensure that the results would be as unaffected as possible by issues related to faulty equipment, misunderstanding of the task or failure to engage with the test, the following exclusion criteria were applied for filtering the data:

- Participants who failed the stereo check during their calibration step (see details on Calibration section) were excluded from analysis
- Participants who produced negative range value—selected a higher JAL than MUL—were excluded from the analysis
- Participants who produced a single value of ranges equal to 0, or 1 across all frequencies, or participants who appeared to have not moved the sliders across all frequencies were excluded from the analysis.
- Participants who submitted twice had their second submission excluded (due to limited number of migraineur group participants taking the test twice).

Table 1 presents the total number and demographic characteristics of the participants in both groups after the filtering was applied using the above criteria.

**TABLE 1.** Participant demographic breakdown

	Control Group	Focus Group
Total number of participants	49	55
Country		
UK + PROLIFIC	27	21
Greece	22	33
Sex		
Female	19	46
Male	30	8
Other/prefer not to say	-	1
Age group		
18–24	6	5
25–44	37	40
45–64	6	10
Official migraine diagnosis		
Yes		46
No		9

Participant demographic breakdown.

published online ahead of print February 15, 2023.

### Stimuli and Test Platform

The stimuli selected for the listening tests were a set of pure tones at the following frequencies: 100, 200, 400, 500, 800, 1000, 2000, 3000, 4000, 6000, 8000, 10,000, and 12,000 Hz. A larger range of frequencies was chosen compared with previous studies, to facilitate a more detailed investigation of hypersensitivity in neighboring frequencies. Very high and very low frequencies were also tested to provide better insight into the participants’ reproduction system’s range and to ensure that a sufficient number of frequencies would be reproduced. The online platform used for the test was Go Listen (Qijian et al. 2021).

### Questionnaire

All participants were requested to fill out a questionnaire before taking the listening test, which was based on the hyperacusis intake questionnaire developed by Dr Richard Tyler at University of Iowa (Richard 2007). The questionnaire was embedded in the platform and consisted of a series of questions determining eligibility for participation (being over 18, no prior hearing loss), demographic information, as well as whether the participant experiences migraines or not, to assign each participant into the migraineur and nonmigraineur group. Participants assigned to the migraineur group were presented with a series of additional questions related to their migraine, such as whether they have received a medical diagnosis or not, severity and frequency of their migraine, and whether they receive any medication for their migraine or not. Finally, migraineur group participants were asked whether they perceive moderately loud sounds as “too loud” compared with others, to determine whether they were aware of hypersensitivity, inquiring more about the characteristics of this phenomenon to those who replied positively. A copy of the full questionnaire can be found in the Supplemental Digital Content, http://links.lww.com/EANDH/B97.

### Calibration

All participants were requested to perform the study using a functional pair of headphones in a quiet room. To ensure safe listening levels as well as maintain a consistent starting volume for all participants, the “rubbing palms” calibration method was employed (Pigeon 2012). More specifically, a prerecorded sample with the sound of palms rubbing against each other was provided to the participants at the stage of system calibration. The sample was recorded using an Earthworks QTC40 microphone, with the palms at a ~5 cm distance from the diaphragm, at the Control Room Studio of Queen Mary University of London. The ambience noise of the room was measured using a Velleman DVM805 sound pressure level meter with a reading of ~30 dB SPL at the time of the recording. The sample was measured from the position of the diaphragm giving readings between 45 and 55 dB SPL on various rubbing intensities. To calibrate their starting listening levels, the participants were asked to match the volume of the prerecorded signal in their headphones with the sound produced by rubbing their palms while holding them ~5 cm away from their nose, taking their headphones off and back on as needed. Upon that the participants were presented with a stereo check, to ensure that their headphones are functional in both ears. More specifically a pure tone was played alternating between the left and right earphone and participants were asked whether they heard a sound coming from the left, right, or both earphones. In addition, participants were asked to note their headphone’s make and model if available, using the comment box provided.

### Main Test

After calibrating their initial listening levels and performing the stereo check, the participants were forwarded to the main test where they were asked to navigate through a series of tones starting from 100 Hz and moving upwards consecutively until they reach 12,000 Hz. Each page included a slider corresponding to a pure tone’s volume at a particular frequency, while pages were grouped in pairs for each frequency, so that participants were requested to first adjust the slider to a position where they can just hear the tone (JAL) and then move to the next page and adjust the slider for the same tone but this time to a level that is perceived as mildly uncomfortable—not painful or causing extreme discomfort. The process was repeated for all 13 pure tones while the participants were requested to only use the given slider on each page to adjust the tone’s volume and not perform any adjustments on their system’s volume after the initial calibration.

### Data Analysis

The two main groups considered in the analysis were the migraineur and nonmigraineur group. For the purpose of the analysis, the migraineur group was divided into further subgroups, based on the following migraine characteristics: migraine phase, migraine frequency, medication, severity. Participants and subgroups as well as subgroup sizes were not matched.

The resulting subgroups are listed below:

- Migraineur on-attack subgroup: participants with migraines who took the test during an attack or aura.
- Migraineur off-attack subgroup: participants with migraine who took the test in their migraine-free, aura-free interval.
- Migraineur on medication subgroup: participants with migraine receiving acute, prophylactic, or combination medication for their migraine.
- Migraineur no medication subgroup: participants with migraine not receiving any medication for their migraine.
- Migraineur low severity subgroup: participants with migraine who rated their migraine’s severity as 30/100 or below.
- Migraineur high severity subgroup: participants with migraine who rated their migraine’s severity as 70/100 or above.
- Migraineur episodic subgroup: participants with migraine who reported experiencing less than 15 attacks per month.
- Migraineur chronic subgroup: participants with migraine who reported experiencing 15 or more attacks per month

To assess our hypothesis that listeners with migraine exhibit smaller dynamic ranges due to lower thresholds of loudness discomfort, we used an α value of 0.05. We initially plotted all responses for both groups in both countries, using a box and whiskers plot to identify the mean, SD, and any potential outliers, as well as look for overlap between the JAL and MUL values, irregularities, or high variability between the responses.

The primary focus of the data analysis was to investigate for signs of decreased dynamic ranges in the migraineur group when compared with the nonmigraineur group, across the range of frequencies assessed in the test. To achieve this, we calculated the ranges between JALs and MULs for each frequency in the two main groups. We then performed a series of two-sample *t* tests (assuming equal variances), for each frequency. Furthermore, the migraineur group was divided into on-attack and off-attack subgroups to test for differences between migraineur group participants that performed the test during their migraine attack/aura and participants who performed the test on their migraine-free, aura-free interval. Average ranges were calculated and plotted across frequencies for the two subgroups, while a two-sample *t* test was performed to assess significance for each frequency. Participants in the migraineur group were also requested to rate their migraine’s severity based on a 101-point numerical rating scale (Williamson & Hoggart 2005).

To determine whether perceived severity is related to decreased sound tolerance, we divided the migraineur group participants into 2 subgroups: participants who rated their migraine’s severity as 30 or below and participants who rated their migraine’s severity as 70 or above. Participants that gave a value between 31 and 69 were excluded from this analysis to avoid bias due to underestimation or overestimation, as well as lack of clarity for values closer to 50.

To investigate the relationship between medication and hypersensitivity, we divided the migraineur group into those receiving medication for their migraine (acute, prophylactic, or both) and those not receiving any medication for their migraine. Finally, we divided migraineur group participants into those with episodic and chronic migraine. The cut off point for participants with chronic migraine was set to 15 or more attacks per month, in accordance with the revised International Classification of Headache Disorders (ICHD-2R) criteria (Bigal et al. 2007).

## RESULTS

**Fig. 1.**
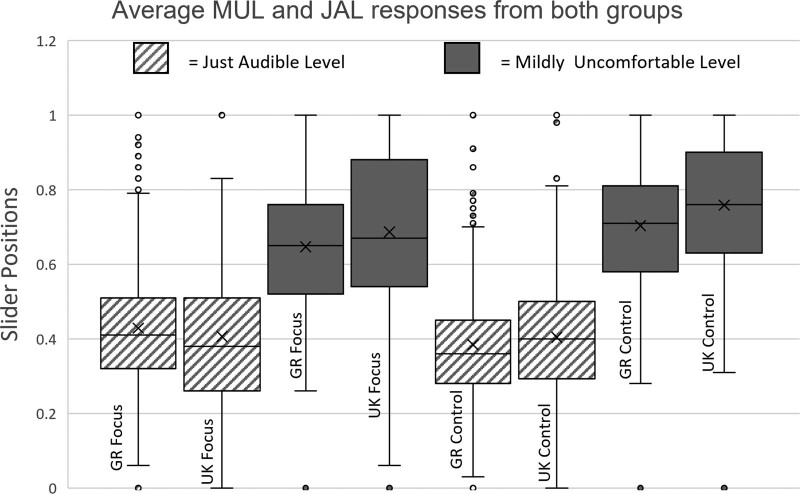
Box and whiskers plot of all responses for JAL and MUL values for the two main groups divided by country and color coded by variable. JAL indicates just audible level; MUL, mildly uncomfortable level.

To visualize the overall participant responses, a box and whiskers plot was produced, which can be seen in Figure 1. Given the observed consistency in JALs across all groups, we normalized the responses of each participant to their own reproduction system and hearing, by using the range between JAL and MUL slider positions as our dependent variable throughout the analysis. This was performed to mitigate the effect of each participants’ unique hearing characteristics, listening setup, and listening conditions on the accuracy of their results.

**Fig. 2.**
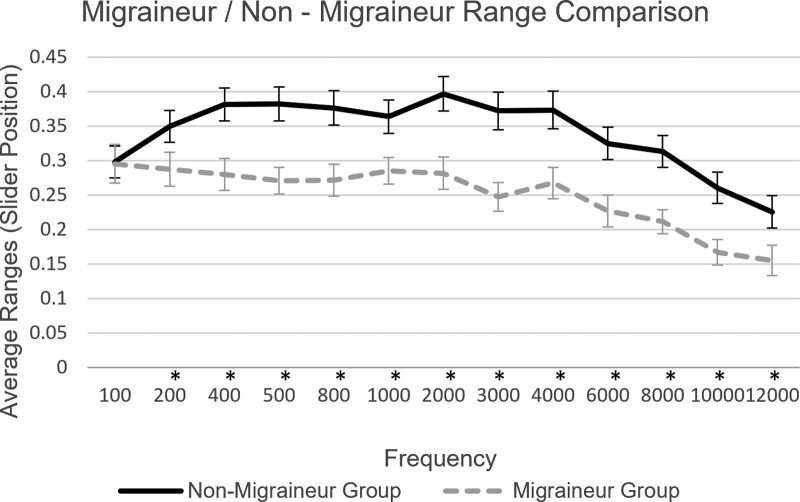
Average ranges between JAL and MUL and SE, plotted over frequency for nonmigraineur group and migraineur group participants (*p < 0.05). JAL indicates just audible level; MUL, mildly uncomfortable level.

Upon plotting the overal responses, the average JAL and MUL ranges were calculated and plotted over frequency for the two main groups, migraineur and nonmigraineur. These can be found in Figure 2. *P* values were also calculated across all frequencies for the two main groups and can be found in Table 2.

**TABLE 2.** *P* values across frequencies

Frequency (Hz)	*p*
100	-
200	*
400	**
500	***
800	**
1000	**
2000	***
3000	***
4000	***
6000	**
8000	***
10,000	***
12,000	*

*P* values across frequencies. Derived through a two-sample *t* test between migraineur and nonmigraineur group on each individual frequency.

-*p* > 0.05; *p < 0.05; **p < 0.01; ***p < 0.001.

**Fig. 3.**
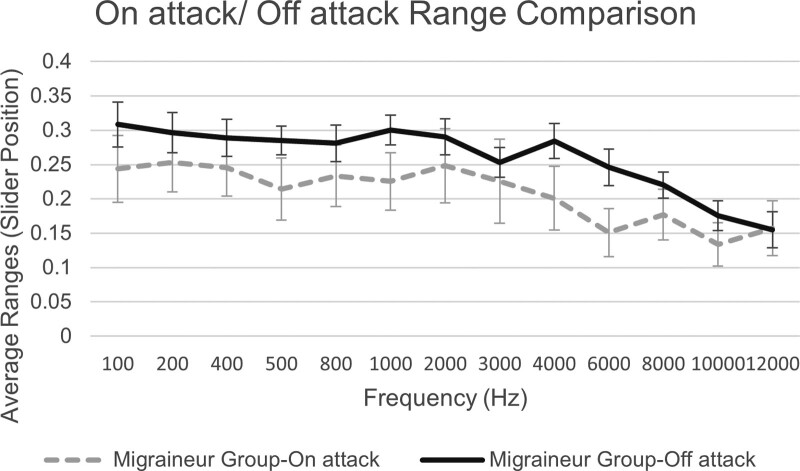
Average ranges between JAL and MUL and SE, plotted over frequency for the on-attack, off-attack migraineur subgroups. JAL indicates just audible level; MUL, mildly uncomfortable level.

Next, on-attack and off-attack subgroup ranges were plotted over frequency. A tendency for the on-attack subgroup to present smaller ranges across most frequencies can be observed in the plot found in Figure 3. Additional *t* test analysis revealed no significant *p* values in the individual frequencies, however a significant *p* value was obtained when the average ranges across all frequencies were tested.

**Fig. 4.**
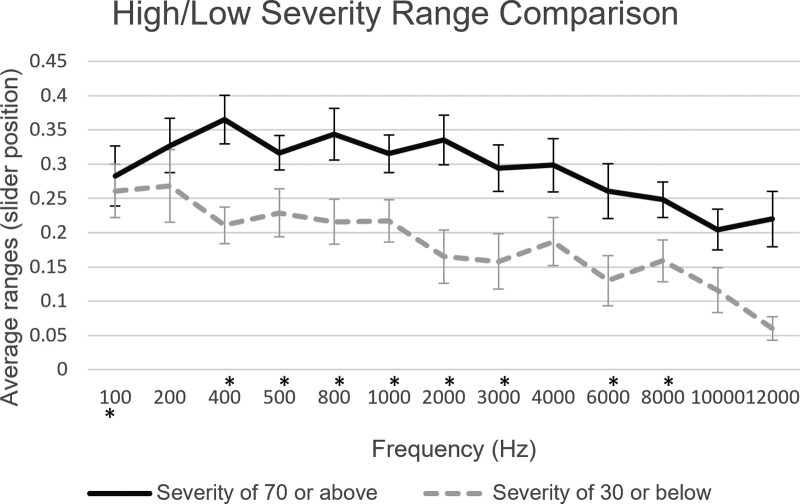
Average ranges between JAL and MUL and SE, plotted over frequency for participants in the high and low severity subgroups (*p < 0.05). JAL indicates just audible level; MUL, mildly uncomfortable level.

Furthermore, average ranges across frequencies exhibited larger values in the self-reported high severity subgroup compared with those in the low severity subgroup, in 9 of 13 frequencies. Plotted values for the subgroups can be seen in Figure 4.

**Fig. 5.**
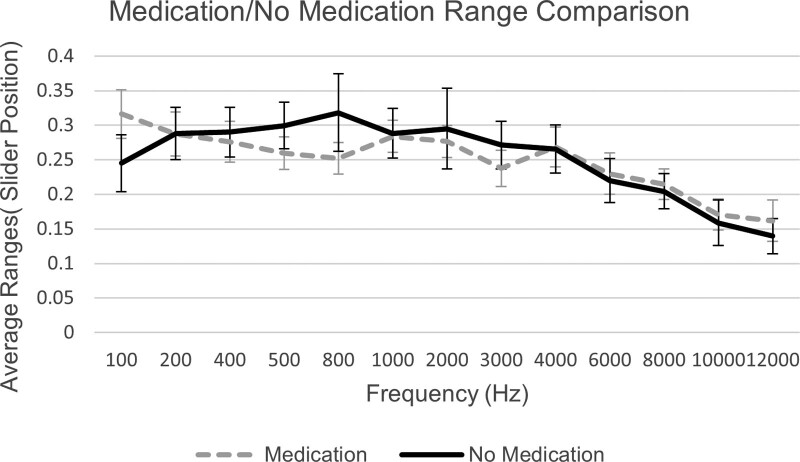
Average ranges between JAL and MUL and SE, plotted over frequency for migraineur participants who receive medication for their migraine and participants who do not. JAL indicates just audible level; MUL, mildly uncomfortable level.

**Fig. 6.**
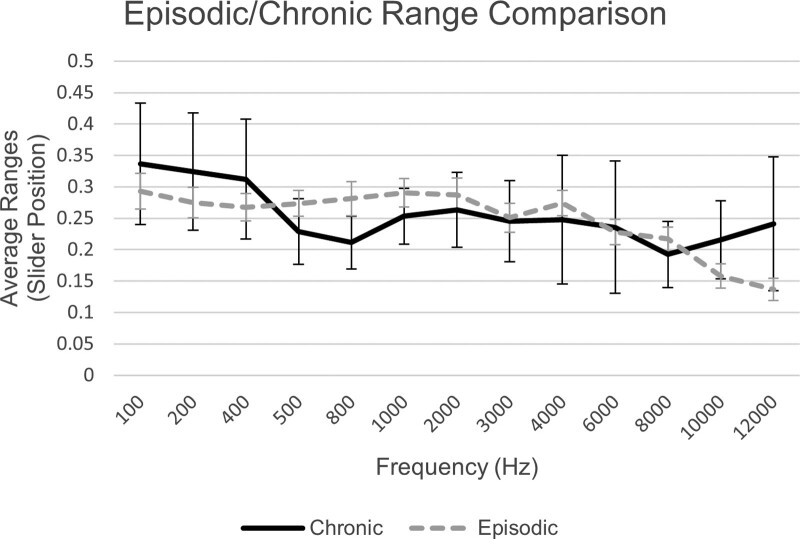
Average ranges between JAL and MUL and SE, plotted over frequency for migraineur participants in the chronic and episodic migraine subgroups. JAL indicates just audible level; MUL, mildly uncomfortable level.

A plot of the average ranges between the subgroups of participants receiving medication for their migraine and not receiving medication, as well as a *t* test across frequency revealed no significant differences between the two. Plotted ranges can be found in Figure 5. Analysis between the average ranges across frequency between the chronic and episodic subgroups also revealed no significant differences. Plotted results can be found in Figure 6.

With regards to demographic observations, comparison of ranges between female and male participants in the migraineur group were not included in the analysis, due to the inequality in the sample size and the risk of bias because the majority of migraineur group participants were female (Table 1). All data and analyses can be found in the Supplemental Digital Content, http://links.lww.com/EANDH/B97.

## DISCUSSION

### Findings

Results demonstrate significantly smaller ranges in the migraineur group participants in 12 of the 13 frequencies tested in the study. The frequencies that appear to be most affected by this phenomenon are those in the mid to high range of the spectrum. Moreover, the mean range values of all the responses for just audible thresholds appear relatively consistent across all groups in both countries and Prolific, thus justifying the attribution of lower dynamic ranges on the variability of the level of mild discomfort, as well as confirming that the participants have followed the calibration instructions to a satisfactory level. Comparison between migraineur participants taking the test during an attack/aura and those taking the test during their migraine/aura-free interval exhibits a tendency for smaller ranges on the on-attack subgroup when plotted over frequency, similarly observed in relevant studies (Ashkenazi et al. 2009) Previous studies have investigated the impact of medication on migraine-related hypersensitivity, observing a positive impact for multimodal prophylactic treatments (Abouzari et al. 2020); however, no significant differences were observed in the medicated versus nonmedicated migraineur group in the analysis herein. This result could be attributed to limitations of self-reporting such as recall bias which could affect the accuracy of the data reported, particularly with regards to the type of medication received, the frequency with which it is received, as well as the lack of information for additional treatments or medication that could be affecting the participants’ hearing. Analysis between migraineur group participants in the self-reported high and low severity subgroups revealed a tendency for the high severity subgroup to present larger ranges in most frequencies, compared with the low severity subgroup participants. This result contradicts relevant studies that observed a positive correlation between migraine intensity and phonophobia (Kelman & Tanis 2006; Yalin et al. 2016); however, irregular results in this study could be attributed to self-report bias and participants’ overestimation/underestimation of their condition. The main result of this study confirms our hypothesis that participants with migraine exhibit lower thresholds of mild discomfort and lower dynamic ranges than participants without migraine and that this phenomenon affects a wide range of frequencies. We observed no evidence associating medication, frequency of migraine attacks, or migraine phase to decreased ranges, however lack of significance in the subgroup analyses, could be due to small sample sizes. The frequencies that appear to be most affected by smaller ranges in this study were those between 400 and 10,000 Hz.

Alhough the exact pathophysiology of sound hypersensitivity in migraine is still under research, results from neuroimaging and neurophysiological studies have demonstrated that migraine patients’ brains demonstrate a hyperresponsiveness to sensory stimuli, with effects such increased attention to stimuli, lack of habituation to repeated stimuli, altered hedonic judgment as well an overall increase in brain activity (Demarquay & Mauguière 2016). The results of this study demonstrate a measurable decrease in the tolerable upper threshold level of auditory stimuli in multiple frequencies, thus underlining the negative impact of the phenomenon in migraineurs’ daily life. Significantly lower thresholds of mild discomfort across most frequencies can impact daily tasks, social activities, work, as well as increase the likelihood of developing depression and anxiety, resulting in an overall reduction in the quality of life of the affected individual (Gokdogan et al. 2021; Pinheiro et al. 2021).

### Impact

Similar to previous studies conducted remotely using either online platforms or telephone surveys (Bexelius et al. 2008; Lipton et al. 2016; Mitsikostas et al. 2018), this study highlights the importance of utilizing web-based platforms, demonstrating their ability to reach and collect data from a wider population. Moreover, the study shows that the utilization of online auditory assessment platforms can be an important tool particularly for longitudinal studies, as well as prescreening, revealing important tendencies and phenomena, and facilitating further research. In addition, this study demonstrates that the phenomenon of hypersensitivity among listeners with migraine extends to a higher range of frequencies than those previously studied. The study also included a diverse population of participants with migraine, from various demographic backgrounds and migraine characteristics, suggesting that hypersensitivity to sound is a common trait among various types of migraine, attack frequencies, and migraine severity in the population. The findings of this study highlight the importance for further research, both toward determining and characterizing the extend of sound hypersensitivity, and the auditory manifestations of migraine in more detail, as well as toward developing specialized treatments and adaptations to alleviate the discomfort caused by this phenomenon. With regards to its clinical relevance, the results of this study further highlight the phenomenon of sound hypersensitivity as well as its extend, thus further research is warranted to investigate whether specific preventive migraine treatments would be of benefit toward mitigating the effects of sound hypersensitivity in migraine sufferers.

### Limitations

Due to its nature and method of delivery, this study could present certain limitations to its accuracy. One such limitation occurs due to measurement error bias, which can develop due to lack of observation, in which case results can be affected by the participants’ equipment, listening space, adherence to guidelines and calibration, as well as their general understanding of the task (Couper 2000). Another limitation of the study is the effect of self-reporting and recall bias, particularly with regards to questions on the severity and frequency of migraine attacks (Althubaiti 2016). Finally, sampling bias is another commonly observed potential limitation in web-based studies. More particularly, in this study, migraineur group participants recruited from migraine support groups and online communities could potentially have little to no familiarity with online listening tests and/or limited technical skills and understanding as well as access to equipment, compared with the nonmigraineur group participants. To mitigate this effect, efforts were made to diversify nonmigraineur group participants, by sharing the test with a wider group of people from various backgrounds in both countries and within the Prolific platform. In addition, the test was designed in a simple way with clear instructions and guidance throughout the process to ensure all participants would be confident to perform the required tasks, regardless of their technical background. Finally, this study does not take into consideration psychosocial factors affecting hypersensitivity, such as association of certain sounds with stressors or uncomfortable environments (Paulin et al. 2019), as well as its effects on the prevalence of this phenomenon in the migraineur population.

### Further Study

To overcome current limitations as well as investigate the phenomenon on a deeper level, future study could include repeating the study in a controlled environment, with the participants using the same calibrated equipment and going through standard audiometry to obtain hearing thresholds before assessing their levels of discomfort. In addition, the study could take place twice for participants with migraine, to assess the impact of migraine phase on the presence of hypersensitivity. A better understanding of the specific frequency characteristics of migraine-related hypersensitivity can aid toward the development of specialized audio adjustment methods, toward improving listening experience for listeners with migraine.

## CONCLUSION

Sound hypersensitivity though well known in the research and clinical community is still an understudied phenomenon that can have a negative impact in the life of a migraine sufferer. Through this study, we have demonstrated that the effects of hypersensitivity can extend to multiple frequencies, reducing sound tolerance for the affected individual. The results of this study highlight the importance of further research both toward a better understanding of the physiological mechanisms of this phenomenon, as well as into effective treatments and adjustments to improve the quality of life of the affected population.

**HIGHLIGHTS**

- Listeners with migraine presented smaller ranges between just audible and mildly uncomfortable sound levels, compared with listeners without migraine.
- Smaller ranges appear in multiple frequencies, with a tendency for the phenomenon to become more prominent when the test is performed during an attack or aura.

## ACKNOWLEDGMENTS

The authors would like to thank Dan Barry, Qijian Zhang, and Andrew Hines of the School of Computer Science University College Dublin, for providing the study with the online platform Go Listen, for the listening tests. We would also like to thank the Migraine Trust, Greek Association of Patients with Migraine and Headache, the migraine and headache online community in Greece as well as Chronic Migraine Awareness UK & ROI - Support Group for their valuable help with participant recruitment.

## Supplementary Material


